# A University-Wide Preparedness Effort in the Alert Phase of COVID-19 Incorporating Community Mental Health and Task-Shifting Strategies: Experience from a Bornean Institute of Higher Learning

**DOI:** 10.4269/ajtmh.20-0458

**Published:** 2020-07-21

**Authors:** Mohamad Hafiz Mukhsam, Mohammad Saffree Jeffree, Nicholas Tze Ping Pang, Syed Sharizman Syed Abdul Rahim, Azizan Omar, Muhammad Syafiq Abdullah, Khamisah Awang Lukman, Nelbon Giloi, Loganathan Salvaraji, Mohd Rahimie Abd Karim, Sahipudin Saupin, Yeap Boon Tat, Mohd Firdaus Mohd Hayati, Mohd Yusof Ibrahim, Assikin Muhamad, Syaza Putri Zainudin

**Affiliations:** 1 Faculty of Medicine and Health Sciences, Universiti Malaysia Sabah, Kota Kinabalu, Malaysia;; 2 Hospital Universiti Malaysia Sabah (HUMS), Universiti Malaysia Sabah, Kota Kinabalu, Malaysia;; 3 Occupational Safety and Health Centre, Universiti Malaysia Sabah, Kota Kinabalu, Malaysia;; 4 Centre for Strategic Management and Corporate Communication, Universiti Malaysia Sabah, Kota Kinabalu, Malaysia

## Abstract

The COVID-19 pandemic caught the world by surprise, causing millions of confirmed cases and hundreds of thousands of deaths. Hence, the Malaysian government announced a Movement Control Order at the start of the containment phase to flatten the epidemiological curve. Universiti Malaysia Sabah (UMS), a public university in Borneo, was accelerated into alert phase because of high risk of case importation from more than 400 China incoming undergraduates. Measures to mitigate the potential COVID-19 outbreaks in its population were taken by using conventional public health measures with special attention to task-shifting and widespread community mental health interventions. A Preparedness and Response Centre was established to overseer the mitigating measures happening inside the university. Measures taken included empowerment of frontline staff, strengthening of restrictions, strengthening university health center, vigorous contact tracing, widespread health education, maintaining cultural sensitivity, and establishment of early standard operating procedures and university continuity plans. Hence, UMS was able to ensure no importation of cases into its campus during both acute and containment phases at the nationwide level.

The COVID-19 virus was first detected in China in late 2019.^1^ On January 30, 2020, the WHO declared COVID-19 as a “Public Health Emergency of International Concern” and later as a pandemic on March11, 2020.^2^ Currently, the WHO has reported more than 3.1 million cases globally, affecting more than 180 countries, with 224,173 COVID-19 fatalities with a reported overall fatality rate of 7.1%.^3^ On January 25, 2020, Malaysia entered the alert phase as it reported its first three confirmed cases of COVID-19 cases, which were importations from a Singapore cluster.^4^ As the number of cases increased, a nationwide Movement Control Order beginning March 18, 2020 was introduced to flatten the epidemiological curve.^5^ Universiti Malaysia Sabah (UMS), a public university on Borneo Island, was one of the organizations that accelerated into preparedness and response during the alert phase. Two potential high-risk factors associated with possible COVID-19 outbreak in the alert phase in UMS were visiting tourists into the university from the ecotourism programme and returning international students from China in early February 2020. Hence, UMS took measures to mitigate the potential fallout of COVID-19 into its population by using conventional public health measures with special attention to widespread community mental health interventions, and task-shifting measures.

An operation centre was established on the first day of the alert phase, chaired by the dean of Faculty of Medicine and Health Sciences and assisted by various coordinators, as shown in Figure 1. The Emergency Operation Centre team served as secretariat for the whole operation, and the Monitoring and Contact Tracing team facilitated rapid isolation and follow-up of Person Under Investigation (PUI) and Person Under Surveillance (PUS) for COVID-19. The Disinfection and Decontamination team coordinated terminal cleaning upon positive case contact, and the Quarantine and Isolation team expedited physical separation of PUI and PUS cases from the public while ensuring their well-being during separation. The Medical Mobile team performed mobile screening and daily surveillance, whereas the Health and Promotion team disseminated continuous health education materials on disinfection, social distancing, and personal hygiene. Stricter policies on screening and testing, usage of personal protective equipment (PPE), return to work, and class attendance were also immediately introduced.

**Figure 1.**
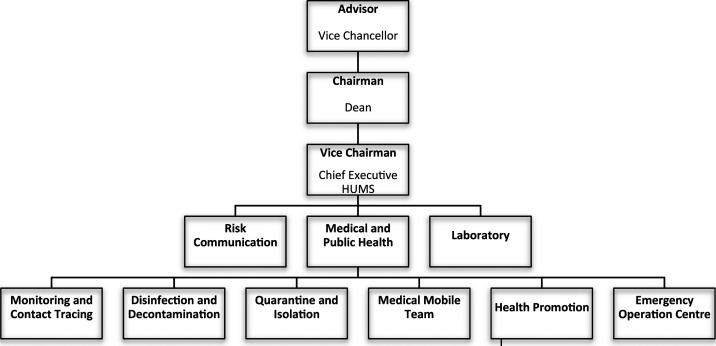
Universiti Malaysia Sabah preparedness and response Centre Committee Organization chart.

Full-fledged situational monitoring, quarantines, and medical screening exercises were initiated in response to the impending return of around 400 Chinese international students. They were home-quarantined for 14 days after initial health screening and only allowed to attend class after medical clearances were given by doctors after home quarantine. During home quarantine, students were divided into several virtual groups of 10 via messaging platforms (WhatsApp or WeChat). Each group was supervised by a local Mandarin-speaking medical student. Each supervisor was tasked with providing psychosocial support and health education, and advising the students to complete daily self-assessment home-monitoring questionnaire using Google Forms. Hence, personal and rapid psychological care was delivered through adoption of a community mental health model described by Bano et al.,^6^ which demonstrably increase bonding and bridging social capital. All information was presented to them in Mandarin, thus breaking the language barrier. Ethnicization has demonstrated efficacy in encouraging health-promotion behaviors.^7^

In-university surveillance was also heightened during the alert phase. Comprehensive data collection during health screening was collated through line listings and shared continuously with local district health officials. Encrypted Google Documents expedited crisis communication, as updates were visible in real time. Through comprehensive surveillance, effective contact tracing was performed. In late January 2020, the team performed contact tracing for 130 individuals (100%) with potential casual contact to a positive COVID-19 case. They were interviewed, screened, and triaged accordingly. This correlates with procedures for diseases with a replication number (*R*_0_) of 2.5 such as COVID-19, whereby more than 70% contact tracing is needed to control an outbreak, and 90% contact tracing is needed to bring down the outbreak.^8^ Throughout the alert phase, only four Chinese students were classified as PUI for COVID-19, but contact tracing was not required as they had been home quarantined compulsorily on arrival. Gate screening was also made compulsory for foreign tourists during the alert phase. They must register at the gate and perform health screening including temperature checks, and prohibitions were initiated at buildings such as the mosque and child health centers to prevent mass infection and protect vulnerable populations. Information was given in several languages, depending on the target population.

The university health centre heightened in importance during the COVID-19 alert phase. In addition to normal daily operation, it was responsible for training of medical professionals, balancing medical equipment and PPE stocks, establishing zones in health centre to minimize exposure to COVID-19 among health professionals, performing health screening and sample taking for COVID-19, and deploying mobile teams for field investigations. Possible issues arising was the backlog of sample processing because of workforce, laboratory equipment, and reagent limitations.^9^ Hence, in the alert phase, UMS proactively procure high amounts of testing kits and reagents, to ready itself for any potential waves of infection and reinfection. In addition, 20 new staff were hired in February and March 2020 to ensure enough staff coverage during potential outbreaks and allow round-the-clock daily operations. Exhaustion, burnout, and physical illness were risks to workforce attrition among frontline staff, including healthcare workers, police, immigration, local governance, and laboratories.^10^ Workload reduction, resilient work schedules, transitioning to electronic health records, teaching mindfulness, and personal coaching are interventions that could promote wellness at workplace.^11^ We introduced a mindfulness-based intervention course for all frontline healthcare workers which was based on an established brief intervention model.^12^ This has the boon of improving psychological mindedness, which has been demonstrated to reduce depressive symptoms through improving dysfunctional coping styles.^13^

Another aspect of mitigating COVID-19 in university is through occupational safety and health. Academicians, students, and other personnel who traveled to COVD-19–affected countries were ordered to undergo health screening and mandatory home quarantine. Guidelines on work or study from home were immediately implemented. Classes were still conducted albeit with several restrictions. Those undergoing attachments outside UMS and symptomatic students and academicians were barred from entering classes. Such policies practiced by UMS was vital, as modeling suggested that workplace closure and social-distancing interventions effectively decreased the rate of infection by 99.3% (interquartile range 92.6–99.99) when *R*_0_ is 1.5, by 93% (interquartile range 81.5–99.7) when *R*_0_ is 2.0, and by 78.2% (59.0–94.4) when *R*_0_ is 2.5.^14^

Moreover, a road show to all 45 faculties and departments was initiated, aiming at immediately disseminating health information and preventative measures. At the same time, these created the perfect platform to carry out workplace risk assessments and improve work procedures to minimize exposure to COVID-19. Appropriate PPE was distributed to frontline staff based on their role and the risk assessment that had been carried out. Personal protective equipment was a vital protection for frontline staff to minimize the risk of exposure to COVID-19 and mortality; hence, required supplies should not be underestimated by both manufacturers and healthcare agencies during anticipated times of crisis.^15^ This is because frontline staff have a significant exposure risk to viruses when in contact with PUI until vaccines are available.^16^ In Wuhan, 63% of the cases were among healthcare workers.^2^ Training of frontline staff according to their role was carried out at the beginning of the alert phase. Tabletop simulations, PPE usage, and clinical and psychological management were some of the topics emphasized during training.

In conclusion, early and effective measures need to be taken during the alert phase, even though it may appear to be the most quiescent. There is high potential of fear and distress during a looming pandemic, and strong measures taken during the alert phase can quell psychological as well as physical contagion.^17^ The entire purpose of the alert phase is to take overarching and far-reaching prevention and anticipation measures, in heightening preparedness of the entire university ecosystem, so that the projected damage or toll to the university in the containment and mitigation phases is minimized. Ideally, all these should begin during peacetime, but UMS was prematurely rushed into an accelerated alert phase because of the sudden necessity to act as more than 400 Chinese students were returning from the heart of the pandemic. Using the mainstays described earlier—strengthening of restrictions, vigorous contact tracing, widespread health education, cultural sensitivity, and establishment of early standard operating procedures and university continuity plans—UMS was able to ensure no importation of cases into UMS campus during both acute and containment phases at the nationwide level.
